# Protective Effect of Astragaloside IV on Hepatic Injury Induced by Iron Overload

**DOI:** 10.1155/2019/3103946

**Published:** 2019-07-25

**Authors:** Dongyu Xie, Ping Zhou, Lin Liu, Wenjing Jiang, Haina Xie, Liang Zhang, Donghao Xie

**Affiliations:** ^1^Department of Spleen-Stomach, Zhenjiang Affiliated Hospital of Nanjing University of Traditional Chinese Medicine, Zhenjiang, China; ^2^Department of Spleen-Stomach, Zhenjiang Hospital of Traditional Chinese Medicine, Zhenjiang, China; ^3^Department of Pharmacy, People's Hospital of Yangzhong City, Zhenjiang, China; ^4^Department of Pharmacy, Dahua Hospital, Xuhui District, Shanghai, China; ^5^School of Basic Medical Science, Shanghai University of Traditional Chinese Medicine, China; ^6^College of Pharmacy, Nanjing University of Chinese Medicine, China; ^7^Department of Pharmacy, Guanghua Hospital of Integrated Traditional Chinese and Western Medicine, Shanghai, China; ^8^School of Pharmacy, Jiangsu University, Zhenjiang, China

## Abstract

Suitable content of iron is essential for human body, but iron overload is associated with many kinds of diseases including chronic liver damage. Recently, researchers find that iron overload promotes hepatocyte autophagy and apoptosis. However, the mechanism of iron overload in liver damage remains unclear. In this study, Lo2 cells were selected as the research object, iron dextran was a model drug, and astragaloside IV was a therapeutic drug to explore the role of iron overload. MTT assay and Annexin/PI double staining were used to measure cell viability and apoptosis. Ultrastructure was observed by transmission electron microscopy. The expression levels of apoptosis and autophagy-related proteins were determined by real-time PCR and Western Blot. The results showed that iron dextran could significantly inhibit Lo2 cell viability and increase the apoptosis rate, while astragaloside IV could reverse the inhibition of Lo2 cell viability and decrease the apoptosis rate. Transmission electron microscopy showed a significant increase in the number of autophagosomes after administration of iron dextran, and the application of astragaloside IV reduced the production of autophagosomes. LC3II/I was significantly upregulated in the model group but decreased in the astragaloside IV treatment group, and P62 showed the opposite trend. Iron dextran significantly upregulated the expression of Bax and downregulated Bcl2, while astragaloside IV reversed this trend. Finally, the inhibition of hepcidin caused by iron dextran was counteracted by astragaloside IV. In conclusion, the experimental results show that the iron overload model mainly induces excessive autophagy and apoptosis of hepatocytes, thus causing damage to hepatocytes, but astragaloside IV plays a certain therapeutic role in reversing this damage.

## 1. Introduction

Iron is one of the basic trace elements necessary for cellular metabolism [1]. The iron content is from 35 to 55 mg Fe/kg body weight in human [2]. The majority of iron in the body is found in hemoglobin, which is used to transport oxygen to other tissues [3]. Under normal circumstances, iron levels in the body are dynamic equilibrium. Therefore, abnormal iron level will cause a series of diseases. For example, the lack of iron can lead to diseases such as iron deficiency anemia [4] and brain dysplasia [5]. Iron overload has a certain link to chronic liver injury [6]. The phenomenon of iron overload usually occurs during some clinical treatments, such as the secondary iron overload associated with conventional transfusion therapy for thalassemia [7]. Iron overload markers also make the disease prognosis worse, with a series of toxic side effects [8].

The mechanism of cell damage and injury causing by iron overload has not been elucidated yet. However, relevant literature shows that iron overload can promote hepatocyte autophagy and apoptosis [9, 10]. Astragaloside is one of the active ingredients of traditional Chinese medicine Astragalus, which has many therapeutic effects, including anti-inflammatory [11] and antirenal fibrosis [12], and can also inhibit hair loss by inhibiting apoptotic signaling [13]. In current study, we found that iron overload could cause liver injury. Then we try to verify the mechanism by which iron overload causes excessive autophagy and apoptosis in liver cells. And we utilize astragaloside as a therapeutic drug, clarifying the mechanism in its protective effect on iron liver injury caused by iron overload from the cell autophagy and apoptosis levels.

## 2. Materials and Methods

### 2.1. Cell Line and Cell Culture

The study was conducted in accordance with the BCPT policy for experimental/preclinical studies [14]. Lo2 cells were derived from normal human hepatocytes and were given by Lu Yin's Lab (Nanjing University of Chinese Medicine). Lo2 cells were cultured with basic DMEM containing 10% FBS (Gibco, Carlsbad, CA, USA) and 1% penicillin/streptomycin (Gibco, Carlsbad, CA, USA) in the atmosphere of 5% CO_2_, 37°C.

### 2.2. MTT Assay

Briefly, Lo2 cells were seeded in 96-well plates at a concentration of 1 × 10^4^ cells/ml and cultured for 12 h in a cell incubator. The modeling drug iron dextran (Sigma, USA) and the therapeutic drug astragaloside IV (Aladdin, Shanghai, China) were diluted with DMEM basic medium. They were inoculated into the corresponding 96-well plates. Lo2 cells added with drug-free media were used as a negative control and cell-free wells served as blank control. The treated cells were incubated in incubator for 24 h. Then 20 *μ*l MTT (5 mg/ml in PBS, Sigma, USA) were added to each well. After 4 h incubation, the supernatant was replaced with 150 *μ*l of DMSO solution. Absorbance OD was measured at 490 nm and cell proliferation was calculated as follows: growth inhibition rate = (1-At/Ac) × 100%; (At, absorbance of the assay; Ac, absorbance of the control) [15].

### 2.3. Transmission Electron Microscopy

Lo2 cells were collected, washed once in PBS, and fixed in 2.5% glutaraldehyde (prepared in PBS) for 2 h. After fixation, cells were rinsed 3 times with 0.1 M phosphoric acid buffer and postfixed with 1% osmium tetroxide solution for 2-3 h. Then the cells were dehydrated in increasing concentrations of ethanol and embedded in Epon-812. LKB-1 ultra-microtome was used to cut the sections of epon-embedded cells into a thickness of 50-60 nm. The sections were double stained with 3% uranyl acetate followed by lead citrate and observed under transmission electron microscope (Japan JEM-1400).

### 2.4. Flow Cytometry (Annexin V + PI Double Staining)

Lo2 cells were seeded into a 6-well plate at the concentration of 1 × 10^5^ cells/ml and then were cocultured with the drugs for 24 h. After that the following operations were carried out. (1) Wash the cells twice with precooled PBS, resuspend the cells in binding buffer (250 *μ*l), and adjust the concentration to 1 × 10^6^ cells/ml. (2) Collect 100 *μ*l cell suspension in 5 ml flow tube and add Annexin V/Alexa Fluor 647 and propidium iodide (PI). Then mix and incubate at room temperature for 15 min (dark condition). (3) Add 400 *μ*l of PBS to the reaction tube and analyze by flow cytometry (BD Biosciences, San Jose, CA, USA).

### 2.5. Immunofluorescence

The Lo2 cells were treated with drugs and cultured as previously described. Then the cells were fixed in 4% paraformaldehyde (precooled, 150 *μ*/well), washed with PBS, blocked with 1% BSA, incubated with primary followed with secondary antibodies, and stained with DAPI in the dark. Immunofluorescence was collected using LSM5 fluorescence microscopy (Zeiss, Germany).

### 2.6. Real-Time PCR

Total RNA of Lo2 cells were extracted using Trizol Reagent Kit (Invitrogen, Carlsbad, CA, USA). cDNA synthesis kit (Promega, Madison, WI, USA) was used to reverse transcribe total RNA. SYBR Green qPCR Mixes (Thermo Fisher Scientific Inc., Grand Island, NY, USA) was used to perform real-time PCR according to the user's manual. This reaction was completed on ABI 7300 system (Applied Biosystems, Foster City, CA, USA). 2^−ΔΔct^ method was used to calculate the expression of target genes and GAPDH was used as endogenous controls. Real-time PCR primers for target genes were as below:

Bax

  Primer F 5′ GCGACTGATGTCCCTGTCTC 3′
  Primer R 5′ GGCCTCAGCCCATCTTCTTC 3′

Bcl-2

  Primer F 5′ GACTTCGCCGAGATGTCCAG 3′
  Primer R 5′ GTGCCGGTTCAGGTACTCAG 3′

P62

  Primer F 5′ GTTCCAGCACAGAGGAGAAG 3′
  Primer R 5′ TGGGAGAGGGACTCAATCAG 3′

LC3

  Primer F 5′ CTCAGACCGGCCTTTCAAGC 3′
  Primer R 5′ TCGTAGATGTCCGCGATGGG 3′

GAPDH

  Primer F 5′ AATCCCATCACCATCTTC 3′
  Primer R 5′ AGGCTGTTGTCATACTTC 3′

*β*-actin

  Primer F 5′ AGGCACTCTTCCAGCCTTCC 3′
  Primer R 5′ CGCCAGACAGCACTGTGTTG 3′

### 2.7. Western Blot

Western blot assay was performed as previously described [16]. Lo2 cells were collected and washed twice with PBS. Then the cells were lysed with RIPA lysis buffer (Solarbio, Beijing, China) containing 1% phosphatase inhibitor and centrifuged at 12000 rpm. Protein concentration was quantified using the Bicinchoninic acid (BCA) protein assay kit (Thermo Fisher Scientific Inc., Grand Island, NY, USA). An equal amount (30 *μ*g) of protein was separated by 10% SDS-PAGE and then was transferred to a nitrocellulose membrane (Millipore Corp., Bedford, MA, USA). 5% skimmed milk powder was formulated with TBST solution containing 1 ‰ Tween-20 and was used to block the membrane for 2 h. The membrane was incubated with primary and secondary antibody. After that the membranes were developed by enhanced chemiluminescence (ECL) kit (Millipore, Burlington, MA, USA). Bands were recorded with Image J and normalized to GAPDH.

### 2.8. Statistical Analysis

All experimental results were represented as mean ± SD. t test was used for the comparison of two sets and ANOVA was used for the comparison of multiple sets. P value was limited to 0.05.

## 3. Results

### 3.1. Astragaloside IV Could Reverse the Decrease of Cell Viability Induced by Dextran Iron

**Figure 1:**
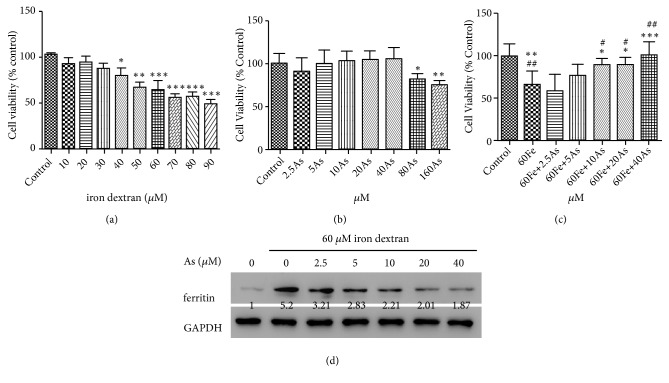
*Astragaloside IV could reverse the decrease of cell activity induced by dextran iron*. MTT experimental results expressed as the relative activity of the drug-administered group and the Control group. (a) Lo2 cells were individually treated with different concentrations of iron dextran. (b) Different concentrations of Astragaloside IV (As) were separately administered to Lo2 cells (DMSO was used as the mother liquid solvent and diluted 1000 times in the single culture), and 1‰ DMSO was added to the control group. (c) Lo2 cells were treated with Fe or/and Astragaloside IV. (d) The expression of ferritin was detected by western blot. The control group was given 1 ‰ DMSO, and the model group was given Fe alone. The treatment group was given different concentrations of Astragaloside IV followed with the 60*μ*M Fe. [*∗* p<0.05, *∗∗* p<0.01, *∗∗∗*p<0.001, VS Control; # p<0.05, ## p<0.01, VS 60Fe].

Iron overload of Lo2 cells was achieved by culturing cells persistently in media containing iron dextran. First, Lo2 cells were treated with 0, 10, 20, 30, 40, 50, 60, 70, 80, and 90 *μ*M iron dextran for 24 h. As shown in Figure 1(a), the half-inhibitory concentration of iron dextran was 63.28 *μ*M. Then, astragaloside IV was used to treat Lo2 cells with the concentrations of 2.5, 5, 10, 20, 40, 80, and 160 *μ*M and was found to be toxic to Lo2 cells at the concentrations of 80 *μ*M (P <0.05) and 160 *μ*M (P <0.01) (Figure 1(b)).

After treatment with 60 *μ*M iron dextran, decreased cell viability was observed (Figure 1(c)). While these effects could be reversed by astragaloside IV in comparison with control, we found that cell viability was significantly restored in astragaloside IV at 10 *μ*M (P <0.05), 20 *μ*M (P <0.05), and 40 *μ*M (P <0.001). Based on these results, Lo2 cells incubated with 60 *μ*M iron dextran and/or 10, 20, and 40 *μ*M astragaloside IV were used in following experiments. Further, we found treatment with 60 *μ*M iron dextran significantly increased the expression level of ferritin, a maker of iron level, while astragaloside IV could attenuate these effects (Figure 1(d)).

### 3.2. Astragaloside IV Reduced the Apoptosis of Lo2 Cells Induced by Iron Overload

**Figure 2:**
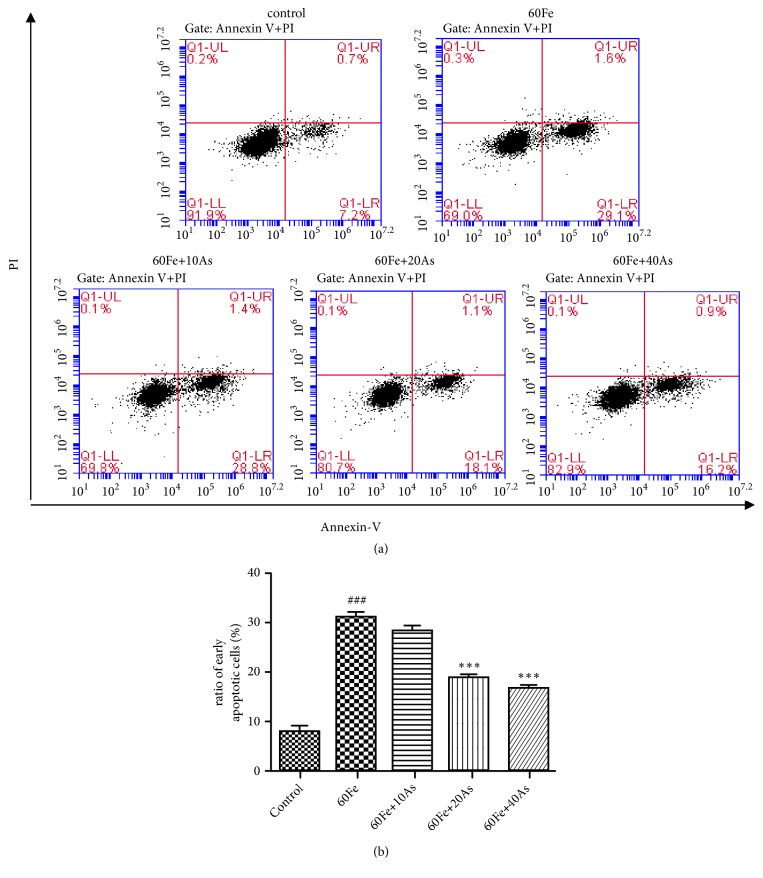
*Astragaloside IV could reduce apoptosis of Lo2 cells induced by iron overload*. (a) Flow cytometry (Annexin V + PI) assay was used to detect the early apoptosis of different treatments in Lo2 cells. (b) The proportion of apoptosis was calculated. The control group was given 1 ‰ DMSO, and the model group was given Fe alone. The treatment group was given different concentrations of Astragaloside IV followed with the 60*μ*M Fe. [###, p<0.001, VS Control; *∗∗∗*, p<0.001, VS 60Fe].

Annexin V + PI double staining kit was used to analyze early apoptotic cells, which were found to be only positive of Annexin V not PI. The proportion of early apoptotic Lo2 cells were screened by flow cytometry (FACS) under different treatments. The experimental results showed that the proportion of early apoptotic cells in the model group (giving 60*μ*M iron dextran) was significantly higher than those in control group (p<0.001). However, the proportion of early apoptotic Lo2 cells in the treatment group (giving 10, 20, and 40*μ*M astragaloside IV followed by 60*μ*M iron dextran) was significantly lower compared with the model group (p<0.001) in a dose-dependent manner (Figure 2). These results suggest that astragaloside IV reduced apoptosis induced by iron overload.

### 3.3. Astragaloside IV Reduced the Autophagy of Lo2 Cells Induced by Iron Overload

**Figure 3:**
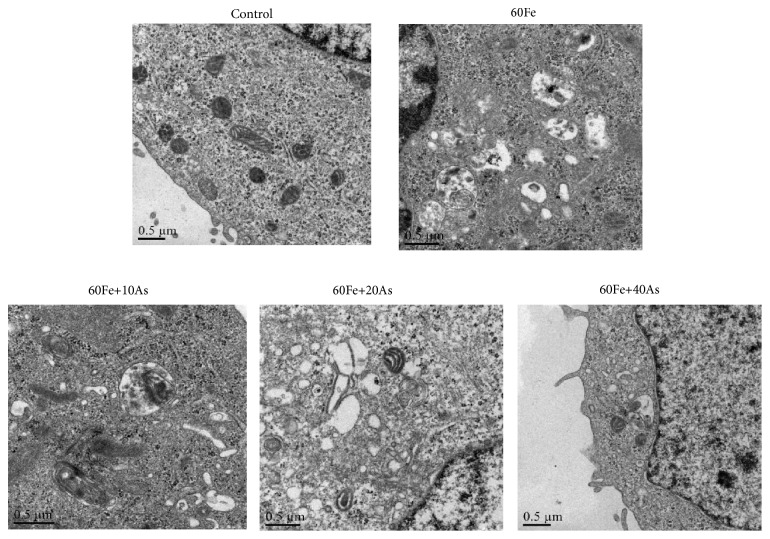
*Astragaloside IV could reduce the increase of autophagosomes induced by iron overload*. Transmission electron microscopy was used to observed autophagosome structure. The shooting multiple was 20000×. There were a large number of autophagosomes in the model group, while the autophagosomes in the control group and the treatment group were rare. The control group was given 1 ‰ DMSO, and the model group was given Fe alone. The treatment group was given different concentrations of Astragaloside IV followed with the 60*μ*M Fe.

**Figure 4:**
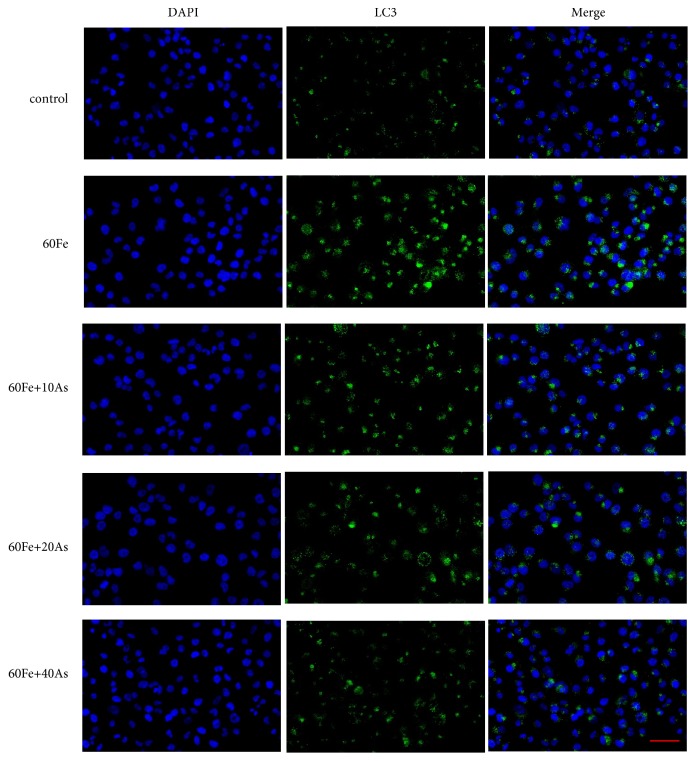
*Immunofluorescence was used to measure the expression pattern of LC3 in Lo2 cells*. Confocal microscope was used to detect the fluorescence. Green fluorescence intensity was positively correlated with LC3 protein expression and blue fluorescence represented the location of the nucleus of each Lo2 cell. The control group was given 1 ‰ DMSO, and the model group was given Fe alone. The treatment group was given different concentrations of Astragaloside IV followed with the 60*μ*M Fe. Scale is 100 *μ*m.

Next transmission electron microscopy was used to observe autophagosomes under different treatment conditions in Lo2 cells. As shown in Figure 3, only a very few cells in control group were observed to have autophagosome-like structure, and the cell morphology was normal. However, a large number of autophagosomes were observed in the model group (giving 60*μ*M iron dextran), suggesting a significant enhancement of autophagy. Excessive autophagy can cause some damage in cell structure, manifested as endoplasmic reticulum, Golgi, and other organelles harm. The astragaloside IV treatment group (given 60*μ*M Fe, followed by 10, 20, and 40*μ*M astragaloside IV) exhibited much fewer autophagosomes in comparison with model group, which was found to be in a dose-dependent manner. Also, cell morphology and structure tended to be normal. Moreover, we investigated autophagy induction after iron dextran and/or astragaloside IV treatment and found that iron dextran induces autophagosome formation, as measured by puncta of LC3 fusion protein in Lo2 cells compared with control group (Figure 4). Together, we supposed that astragaloside IV may play a therapeutic role in iron overload liver injury.

### 3.4. Astragaloside IV Attenuated Iron-Induced Apoptosis and Autophagy via Regulating Hepcidin in Lo2 Cells

**Figure 5:**
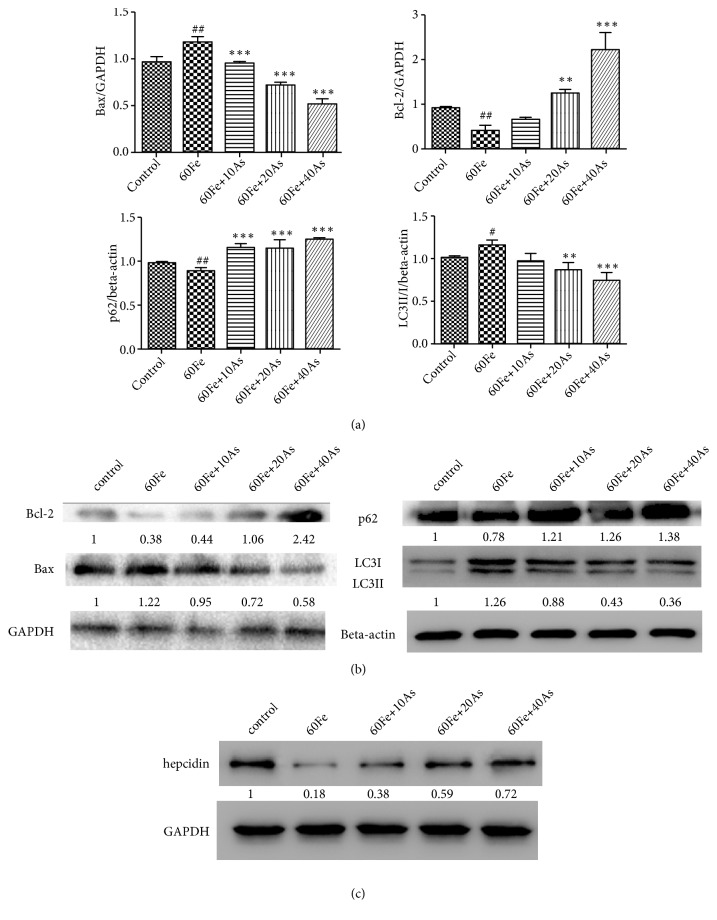
*Astragaloside IV could reduce the apoptosis and the excessive autophagy in iron-induced Lo2 cells at the protein level*. The expression levels of Bax, Bcl-2, p62 and LC3II / I were determined by real-time PCR (a) and western blot (b). (c) The expression levels of hepcidin were detected by western blot. The control group was given 1 ‰ DMSO, and the model group was given Fe alone. The treatment group was given different concentrations of Astragaloside IV followed with the 60*μ*M Fe. [#, p<0.05, ##, p<0.01, VS Control; *∗∗*, p<0.01, *∗∗∗*, p<0.001, VS 60Fe].

Further, we wanted to explore the mechanism involved in this process. As we found that iron overload induced cell apoptosis, we detected apoptosis related makers and found that cells treated with 60*μ*M Fe exhibited higher level of Bax and lower level of Bcl2 in comparison with control. The cells treated with 60*μ*M Fe and astragaloside IV showed lower level of Bax and higher level of Bcl2 in comparison with the cells treated with only 60*μ*M Fe (Figures 5(a) and 5(b)). These data suggested that Bax/Bcl-2 pathway was involved in this process.

Then the levels of p62 and LC3II / I were measured by western blot and real-time PCR. As shown in Figures 5(a) and 5(b), we found that, compared with control group, the expression of LC3II / I was increased and the expression of p62 was significantly decreased in Lo2 cells (P <0.01) in which 60*μ*M iron dextran was applied. It was demonstrated that iron dextran induces autophagy in hepatocytes. After applying different concentrations of astragaloside IV, the expression of p62 was significantly increased (p <0.001), and LC3II/I was significantly decreased (p<0.001). It means that astragaloside IV could significantly attenuate the overautophagy phenomenon in model group.

In addition, Hepcidin is a well known regulator of iron and controls iron release from cells that recycle or store iron, thus regulating plasma iron concentrations [3]. The decrease of Hepcidin was found in the model group (60*μ*M Fe) compared with control group, while astragaloside IV increased the expression level of Hepcidin in a dose-dependent manner compared with model group (Figure 5(c)). These results indicated that astragaloside IV attenuated iron-induced apoptosis and autophagy via upregulating hepcidin.

## 4. Discussion

Iron is essential for almost all organisms and is an integral part of many metabolic functions [17]. Iron has a wide range of physiological functions and is closely related to the synthesis of hemoglobin and myoglobin. It also serves as a component in essential enzyme for the body [18]. The more iron body accumulates, the more energy is released. Therefore, iron is particularly likely to accumulate in the heart, liver, kidney, and other organs with a high degree of physiological activity and biochemical functions. The level of iron tends to maintain a state of dynamic balance in the body. Abnormality of iron level will lead to a series of diseases.

Clinically, conventional blood transfusions are the best means of treating many diseases of the blood system. However, iron overload is an inevitable consequence of transfusions and is often accompanied by an increase in iron absorption from the gut [19]. Iron, on the other hand, is most likely to accumulate in energetically metabolized liver [20], so patients are prone to cause liver damage during long-term massive blood transfusions. Astragalus, as a Chinese medicine, has the effects of Buqi, diuretic swelling and promoting wound healing [21]. And astragaloside astragalus is one of the active ingredients. Therefore, it has important clinical significance and value to study the protective effect of astragaloside on liver injury and to explore its protective mechanism.

In this study, MTT test showed that giving a certain amount of iron can reduce the activity of hepatocytes. With the application of effective and nontoxic dose of astragaloside IV, liver cell vitality has been restored. This study initially confirmed that astragaloside IV could reverse the decline in viability of iron overload cells. According to the relevant literature which have reported that liver injury is caused by iron overload and induction of excessive autophagy and apoptosis in liver cells [8, 9], therefore, we observed a large number of autophagosomes in iron overload cells using transmission electron microscopy, while the number of autophagosomes was significantly reduced after treatment with astragaloside IV. Next, we used flow cytometry to detect hepatocyte apoptosis and found that the proportion of Annexin V (PI-) hepatocytes was significantly increased in iron overload cells, while the proportion was decreased after giving astragaloside IV. Finally, the expression levels of autophagy and apoptosis-related proteins were measured by immunofluorescence assay, real-time PCR, and western blot in iron overload model group and the astragaloside IV treatment group. Our results showed that LC3II / I was upregulated and p62 was downregulated in iron overload cells, while these effects could be reversed by astragaloside IV. LC3 and p62 levels are often used to measure autophagy which is a common cellular survival mechanism [22]. Degradation of p62 and reduction in LC3-I mean that autophagy occurs [23]. In this study, when Lo2 cells were exposed to iron overload, the levels of LC3-II/ LC3-I increased and those of p62 decreased. So we concluded that autophagy was activated by iron overload and astragaloside IV was found to alleviate autophagy. Moreover, we found that the expression level of Bax was increased and Bcl-2 was decreased in iron overload cells, while astragaloside IV could reverse these effects. Finally, we found that the expression of Hepcidin was inhibited by iron overload, but astragaloside IV could increase Hepcidin in a dose-dependent manner. However, the deeper mechanism of the role of astragaloside IV needs to be explored in the following study.

## 5. Conclusions

In summary, this study initially demonstrated the protective effect of astragaloside against iron overload model at the cellular level, which is related to the inhibition of excessive autophagy and apoptosis of iron overload hepatocytes.

## Data Availability

The data used to support the findings of this study are available from the corresponding author upon request.
